# 57084 Combining artificial intelligence and robotics: a novel fully automated optical coherence tomography-based approach for eye disease screening

**DOI:** 10.1017/cts.2021.712

**Published:** 2021-03-30

**Authors:** Ailin Song, Pablo Ortiz, Mark Draelos, Stefanie G. Schuman, Glenn J. Jaffe, Sina Farsiu, Joseph A. Izatt, Ryan P. McNabb, Anthony N. Kuo

**Affiliations:** 1 Duke University School of Medicine; 2 Duke University, Department of Biomedical Engineering; 3 Duke University, Department of Ophthalmology

## Abstract

ABSTRACT IMPACT: Despite its importance in systemic diseases such as diabetes, the eye is notably difficult to examine for non-specialists; this study introduces a fully automated approach for eye disease screening, coupling a deep learning algorithm with a robotically-aligned optical coherence tomography system to improve eye care in non-ophthalmology settings. OBJECTIVES/GOALS: This study aims to develop and test a deep learning (DL) method to classify images acquired from a robotically-aligned optical coherence tomography (OCT) system as normal vs. abnormal. The long-term goal of our study is to integrate artificial intelligence and robotic eye imaging to fully automate eye disease screening in diverse clinical settings. METHODS/STUDY POPULATION: Between August and October 2020, patients seen at the Duke Eye Center and healthy volunteers (age ≥18) were imaged with a custom, robotically-aligned OCT (RAOCT) system following routine eye exam. Using transfer learning, we adapted a preexisting convolutional neural network to train a DL algorithm to classify OCT images as normal vs. abnormal. The model was trained and validated on two publicly available OCT datasets and two of our own RAOCT volumes. For external testing, the top-performing model based on validation was applied to a representative averaged B-scan from each of the remaining RAOCT volumes. The model’s performance was evaluated against a reference standard of clinical diagnoses by retina specialists. Saliency maps were created to visualize the areas contributing most to the model predictions. RESULTS/ANTICIPATED RESULTS: The training and validation datasets included 87,697 OCT images, of which 59,743 were abnormal. The top-performing DL model had a training accuracy of 96% and a validation accuracy of 99%. For external testing, 43 eyes of 27 subjects were imaged with the robotically-aligned OCT system. Compared to clinical diagnoses, the model correctly labeled 18 out of 22 normal averaged B-scans and 18 out of 21 abnormal averaged B-scans. Overall, in the testing set, the model had an AUC for the detection of pathology of 0.92, an accuracy of 84%, a sensitivity of 86%, and a specificity of 82%. For the correctly predicted scans, saliency maps identified the areas contributing most to the DL algorithm’s predictions, which matched the regions of greatest clinical importance. DISCUSSION/SIGNIFICANCE OF FINDINGS: This is the first study to develop and apply a DL model to images acquired from a self-aligning OCT system, demonstrating the potential of integrating DL and robotic eye imaging to automate eye disease screening. We are working to translate this technology for use in emergency departments and primary care, where it will have the greatest impact.

